
JOURNAL INFORMATION
==============================
NLM Title Abbreviation: ScientificWorldJournal
Journal ID: ScientificWorldJournal
NLM Journal ID: 101131163
Journal ID: TSWJ

The Scientific World Journal

ISSN: 2356-6140
EISSN: 1537-744X
Publisher: Wiley

ARTICLE INFORMATION
==============================
PMCID: PMC5956365
PMID: 15349543
DOI: 10.1100/tsw.2004.65
Article ID: 350127
Article version: 1
Subjects: Case Report

A Rare Condition: The Ureteritis Cystica

Kýlýç Süleyman 1
Sargin Semih Yaşar 3
Günes Ali 1
Ipek Deniz 1
Baydinç Can 1
Altinok M. Tayfun 2
1 Department of Urology, Inonu Universitesi Tip Fakultesi, Turgut Ozal Tip Merkezi, Uroloji AD, Elazig Yolu 9. Km, 44069, Malatya, Turkiye
2 Departments of Radiology Inonu Universitesi Tip Fakultesi, Turgut Ozal Tip Merkezi, Uroloji AD, Elazig Yolu 9. Km, 44069, Malatya, Turkiye
3 Department of Urology, Yüksek İhtisas Hospital, Ankara, Turkey
Academic Editor: Anthony Atala

Publication date: 2004
Electronic publication date: 2004 Jun 7
Volume: 4
First page: 175
Last page: 178
Copyright: Copyright © 2004 Süleyman Kýlýç et al.
Copyright year: 2004
License: This is an open access article distributed under the Creative Commons Attribution License, which permits unrestricted use, distribution, and reproduction in any medium, provided the original work is properly cited.
License URL: https://creativecommons.org/licenses/by/3.0/

==============================
Case Report
*TheScientificWorldJOURNAL* (2004) 4 (S1), 175–178
ISSN 1537-744X; DOI 10.1100/tsw.2004.65

### A Rare Condition: The Ureteritis Cystica

Süleyman Kýlýç[1], Semih Yaşar Sargin[3], Ali Günes[1], Deniz Ipek[1], Can Baydinç[1], and M. Tayfun Altinok[2]

*Departments of Urology[1] and Radiology[2]; Inonu Universitesi Tip Fakultesi, Turgut Ozal Tip Merkezi, Uroloji AD, Elazig Yolu 9. Km, 44069, Malatya, Turkiye; [3]Department of Urology, Yüksek İhtisas Hospital, Ankara, Turkey*

**E-mails**: skilic@inonu.edu.tr; drskilic@hotmail.com

*Previously published in the Digital Urology Journal*

---

**DOMAIN**: urology

---

### CASE PRESENTATIONS

### Case One

In November 1997, a 65-year-old woman was admitted with a complaint of stress urinary incontinence for 2 years. Dysuria, hematuria, and any systemic illness were not noted in her medical history. Physical examination revealed only grade-2 cystocele. Bonney and cotton swab tests were positive. 8-10 erythrocytes and 2-3 leucocytes per high-power field were detected by urine analysis. No bacterial growth was established at midstream urine culture. Blood levels of urea, creatinine, uric acid, and electrolytes were within normal limits. The ultrasonography (USG) of the kidneys and bladder was normal. IVP showed 3 and 4 filling defects in the left and right ureters respectively (Figures 1 and 2). A computerized tomography of the abdomen and pelvis demonstrated an intraluminal lesion in the proximal part of the right ureter that covered the lumen incompletely. A multichannel cystometry confirmed the pure stress incontinence. Cytology findings of selective urine specimens collected from both ureters under local anesthesia were negative for atypical cells. Bilateral rigid ureteroscopies were performed under general anesthesia. Six polypoid lesion in the right and 4 lesions in the left ureter were diagnosed during this procedure. Multiple biopsies were done bilaterally and laparoscopic retropubic bladder neck suspension was performed subsequently to correct the stress incontinence. Histopathologic examinations of the biopsy specimens were negative for malignancy and the condition was diagnosed as bilateral UC. The patient was followed every 6 or 12 months for a period of 45 months. Routine contrast and cytology studies showed no evidence of malignancy and no significant interval changes in the lesions, ureters, and intrarenal collecting systems.

### Case Two

In March 2000, a 45-year-old man who had been operated on for chronic otitis media by the otorhinolaryngology clinic was seen in consultation by the urology clinic for symptoms of bladder outlet

TheScientificWorldJOURNAL (2004) 4(S1), 175–178

obstruction. He had a history of intermittent macroscopic hematuria and a left nephrolithotomy operation (1987). Suprapubic pain was noted on physical examination. A urine microscopy demonstrated 9-10

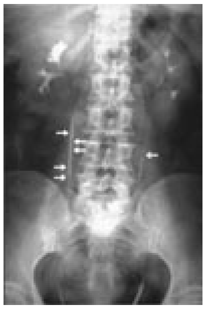

**FIGURE 1**. IVP showing the filling defects in the right ureter of first patient.

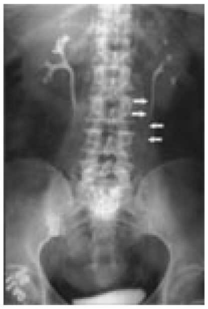

**FIGURE 2.** IVP showing the filling defects in the left ureter of first patient.

erythrocytes and 4-5 leucocytes per high-power field. A urine culture diagnosed E.coli greater than 100,000 colonies per milliliter of urine. Appropriate antibiotic therapy was started. Blood levels of urea, creatinine, electrolytes, and white cells were in normal ranges. USG revealed bilateral hydronephrosis, one stone in the right kidney, and one stone in the bladder. Additionally, a small filling defect was demonstrated on the right side of the bladder wall on the IVP (Figure 3). Diuretic renogram revealed nonobstructive residual dilatation in the left kidney and obstructive dilatation in the right. A polypoid lesion, approximately 8 mm. in size, in the right ureteral orifice was observed during cystoscopic examination under general anesthesia. It was resected and pneumatic lithotripsy for the bladder stone at lithotomy position and right nephrolithotomy at flank position were subsequently performed. Histopathologic examination of the lesion defined the UC. Cystoscopy was performed every 6 months for 20 months and no recurrence or cancer development has been established to date.

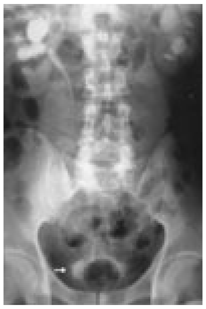

**FIGURE 3**. A filling defect on the right side of bladder in second patient.

### DISCUSSION

UC was first described by Richmond and Robb.(1) This pathology more commonly affects older people and women and may be bilateral. The most accepted cause is urinary infection. In 1997, Menendez et al published a review of 34 patients.(2) In this series infection was reported in 53% of the cases. Infection was defined in one of the present cases. Some other causes have also been reported. For example, UC developed in an oncology patient following instillation of formalin for the treatment of cyclophosphamide-induced hemorrhagic cystitis.(3)

UC is a silent pathology without any specific symptoms. Sometimes obstruction secondary to the lesions and scarring associated with the long-standing infection may lead to pain. As in the present cases, UC may be diagnosed incidentally during the evaluation of different conditions. A suspicious renal mass was found in the examination of the urinary system of a hypertensive patient and the histopathology of the surgical specimen demonstrated UC.(4)

Imaging techniques such as IVP and RGP are gold standard methods in the diagnosis and follow-up of UC. Small filling defects and a bead-like appearance with regular surfaces in the ureter and renal pelvis are the typical findings demonstrated in these examinations. Magnetic resonance urography can provide high-resolution of coronal images of the entire urinary tract without using contrast agents and ionizing radiation. A major concern is the cost of the procedure. UC may also be identified and removed successfully during a ureterorenoscopy.

Although UC and carcinoma may occur together in the same patient, UC does not appear to be a premalignant lesion. In the literature, there is only one case of the UC concomitant with the adenocarcinoma of the ureter.(1) However, this patient had no prior radiological evaluations and it would seem that the UC was only an incidental finding. Some authors believe that cell nests can assume mucus secreting properties and cystic structures of the UC represent metaplastic changes of the transitional epithelium due to the chronic inflammation or other causes of the mucosal irritation. They may regress completely if the underlying pathogenetic factor is removed. If not, patients with extensive metaplasia are in a high risk for the development of adenocarcinoma.(5,6) In one report, a patient with a 17 year follow-up, who had transitional cell carcinoma of the bladder and UC in the ureter of a solitary kidney, was reported.(7) There was no appreciable radiological change in characteristic ureteropyelographic appearance and no carcinoma recurrence. Kindall reported a patient with a 6.5 year follow-up where no evidence of the cancer was found.(8) Also, no cancer development was observed in the present patients in 2 and 4 year follow-ups. From these findings, we believe that UC is a benign pathology with indolent evolution and variable duration.

Various therapies can be applied in the treatment of UC. In 1946, Kopp obtained good results from the instillation of 2% silver nitrate into the ureters.(9) Petersen recommended a conservative attitude using long-term antibiotics until normal radiography findings were obtained.(10) However, if infection is not present, this measure seems unwarranted and side effects related to antibiotics may arise. Patients in the present report, and a patient in another case report,(7) received no additional treatment and there was no distortion in their conditions.

### CONCLUSIONS

In conclusion, UC is a benign inflammatory pathology; even though it may mimic a malign tumor with radiography findings. It has a benign course with rare complication and progression. We recommend follow-up if the patient has no any urinary infection or complication(s) secondary to UC.

### REFERENCES

1. Richmond HG, Robb WAT: Adenocarcinoma of the ureter secondary to ureteritis cystica. Brit J Urol, 39:359, 1967.

2. Menendez V, Sala X, Alvarez-Vijande R, Sole M, Rodriguez A, Carretero P: Cystic pyeloureteritis. Review of 34 cases. Radiological aspects and differential diagnosis. Urology, 50(1):31, 1997.
3. Mahboubi S, Duckett JN, Spackman TJ: Ureteritis cystica after treatment of cyclophosphamide-induced hemorrhagic cystitis. Urology, 7:521, 1976.
4. Askari A, Herrera HH: Pyeloureteritis cystica. Urology, 16(4):398, 1980.
5. Ward AM: Glandular neoplasia within the urinary tract. The aetiology of adenocarcinoma of the urothelium with a review of the literature. I: Introduction: the origin of glandular epithelium in the renal pelvis, ureter and bladder. Virchows Arch A, 352:296, 1971.
6. Kashgarian M, Rosai J: Urinary tract. In: Ackermanís Surgical Pathology. Rosai J, editor. St.Louis: CV Mosby Comp; p. 819-922, 1988.
7. Duffin TK, Regan JB, Hernandez-Graulau JM: Ureteritis cystica with 17-year followup. J Urol, 151:142, 1994.
8. Kindal L: Pyelitis cystica and ureteritis cystica: Report of a case diagnosed by urography and confirmed by biopsy, with an outline of treatment. J Urol, 29:645, 1933.
9. Kopp JH: Pyelitis, ureteritis and cystitis cystica. J Urol, 56:28, 1946.
10. Petersen UE, Kvist E, Friis M, Krogh J: Ureteritis cystica. Scand J Urol Nephrol, 25(1):1, 1991.

**This article should be referenced as follows:**

Kilic, S. (2004) A rare condition: the ureteritis cystica. *TheScientificWorldJOURNAL* **4 (S1)**, 175–178.

**Handling Editor:**

Anthony Atala, Principle Editor for *Urology* — a domain of *TheScientificWorldJOURNAL*.
